# Supplementary material for: Intention to use mobile health in antenatal care service: Among primary health care unit workers, Bench-Sheko zone, southwest Ethiopia
Source: PLOS Digit Health. 2025 Apr 22;4(4):e0000587. doi: 10.1371/journal.pdig.0000587 (PMC12013934; doi:10.1371/journal.pdig.0000587)
Supplement: Attachment — Submitted filename: PLOS DIG-D-24-00289 - Reviewer Borges, Julian YV.pdf [file pdig.0000587.s001.pdf]

Intention to Use Mobile Health Technology in Antenatal Care Services Among Primary Health Care Unit Workers in Bench-Sheko Zone, Southwest Ethiopia

Reviewer Report - DIG-D-24-00289

Overview

The manuscript examines the intention of primary health care unit (PHCU) workers to use mobile health (mHealth) technology for antenatal care (ANC) services in Bench-Sheko Zone, Southwest Ethiopia. It addresses an important topic in the field of maternal health services in low- and middle-income countries. The study uses a cross-sectional design with a sample size of 316 PHCU workers and employs multivariable linear regression modeling for data analysis. The findings highlight the importance of perceived mobile self-efficacy, perceived compatibility, mobile ownership, eHealth training, and mobile use experience as significant predictors of behavioral intention to use mHealth technology in ANC services.

Major Issues:

1. Theoretical Framework:
    - The theoretical framework is not clearly described. The authors should provide more information on the specific behavioral theories and technology acceptance models used and how they relate to the study's objectives and hypotheses.
2. Generalizability:
    - The generalizability of the findings may be limited due to the focus on a single geographical area. The authors should discuss this limitation and provide more information on the characteristics of the PHCU workers who participated in the study.
3. Questionnaire Validation:
    - The validation process for the questionnaire is not described in sufficient detail. The authors should provide more information on the specific validation methods used, the results of the validation, and any adaptations made to the questionnaire for the current study.
4. Discussion Section:
    - Strengthen the discussion by comparing the study's findings with those of similar studies in other low- and middle-income countries.
    - Discuss any unexpected findings and provide potential explanations for these results.
5. Conclusion and Recommendations:
    - Provide more actionable and specific recommendations for future research, such as longitudinal studies and mixed-methods approaches.
    - Discuss practical implementation of findings in the Ethiopian healthcare context.
6. Data Availability:
    - Provide information on data availability and any restrictions on access.

Minor Issues:

1. Abstract:
    - Include more specific statistical results for clarity.
    - Ensure consistency in reporting results.
2. References:
    - Ensure all references are complete, relevant, and formatted according to the journal's guidelines.
3. Formatting:
    - Ensure consistency in headings and subheadings.
    - Check for minor typographical errors and grammatical issues throughout the manuscript.
4. Ethical Considerations:
    - Add a statement on how participants' confidentiality and data security were ensured during the study.

Summary

The manuscript explores the intention of primary health care unit (PHCU) workers to adopt mobile health (mHealth) technology for antenatal care (ANC) services in Bench-Sheko Zone, Southwest Ethiopia. It addresses a crucial topic in maternal health, particularly in low- and middle-income countries, and provides valuable insights into key predictors of mHealth adoption. However, several areas require significant improvement.

The theoretical framework needs clarification, specifically detailing the behavioral theories and technology acceptance models employed. The study's generalizability is limited by its focus on a single geographical area, which should be discussed. More information is needed on the validation process of the questionnaire used.

The discussion section should be strengthened by comparing findings with similar studies in other regions and discussing any unexpected results. The conclusion and recommendations should offer more actionable insights for future research and practical implementation in the Ethiopian healthcare context. Additionally, the manuscript should provide information on data availability.

Minor issues include ensuring statistical clarity in the abstract, complete and properly formatted references, consistent formatting throughout, and a statement on ethical considerations regarding participant confidentiality and data security.

Other comments: The study addresses an important gap in the literature and uses appropriate methods for data collection and analysis. The high response rate (98.7%) is a strength of the study. However, the authors should consider the limitations of self-reported data and the potential for response bias.

Figures and Tables: Yes, but demographic data should be presented in a table for better readability.

Methods: The methods section provides sufficient detail on the study design, sample size, sampling technique, data collection tools and procedures, and data analysis. However, the authors should provide more information on the validation process for the questionnaire used in the study.

Study Design: The cross-sectional study design is appropriate for assessing the intention of PHCU workers to use mHealth technology in ANC services. However, the authors should acknowledge the limitations of this design in establishing causal relationships between variables.

Any Experiments Missing? The study does not involve any experiments, as it is a cross-sectional survey.

Statistics: The statistical analyses used in the study, including multivariable linear regression modeling, are appropriate for the research question and the nature of the data collected. However, more detailed information on the regression analysis is required.

Results and Data: The results and data presented in the manuscript support the conclusions drawn by the authors. The significant predictors of behavioral intention to use mHealth technology in ANC services are identified and discussed but more detailed presentation and analysis are needed.

All data accessible? The manuscript does not mention whether the data used in the study are accessible. The authors should provide information on data availability and any restrictions on access.

References: The references appear to be appropriate and relevant to the study. However, the authors should double-check the references to ensure that they are complete and formatted according to the target journal's guidelines.

Title: The title is appropriate and informative, clearly stating the study's focus on the intention to use mHealth technology in ANC services among PHCU workers in a specific region of Ethiopia.

Conclusion

In conclusion, the manuscript addresses an important topic and uses appropriate methods for data collection and analysis. However, there are some areas that require further clarification and improvement, particularly regarding the theoretical framework, generalizability of the findings, and validation of the questionnaire. The authors should address these issues before the manuscript can be considered for publication.
