# Supplementary material for: Intention to use mobile health in antenatal care service: Among primary health care unit workers, Bench-Sheko zone, southwest Ethiopia
Source: PLOS Digit Health. 2025 Apr 22;4(4):e0000587. doi: 10.1371/journal.pdig.0000587 (PMC12013934; doi:10.1371/journal.pdig.0000587)
Supplement: Attachment — Submitted filename: Response to reviewers.docx [file pdig.0000587.s003.docx]

Shimeles Wondimu Kebede

Mizan-Aman Health Science College, Department Of Public Health

Mizan-Aman City, Bench-Sheko Zone, South West Ethiopia

[Shimeleswondimu@Gmail.Com](mailto:shimeleswondimu@gmail.com)

Date- October 9, 2024 G.C

PLOS Digital Health

Dear Reviewer’s

Thank you for your thoughtful and constructive feedback on our manuscript titled **"Intention to Use Mobile Health Technology in Antenatal Care Services: Among Primary Health Care Unit Workers in Benchsheko Zone Southwest Ethiopia."** We appreciate the time and effort you dedicated to reviewing our work. We have carefully considered your comments and have made revisions to address each concern. Below, we detail our responses:

**Reviewer #1's Comments and Our Responses**

- **Comment 1: - Theoretical Framework** Lacks clarity. Needs a better description, including specific behavioral theories and technology acceptance models.
- **Response 1:-** We have enhanced the description of the theoretical framework by incorporating specific behavioral theories and technology acceptance models, including the Technology Acceptance Model (TAM), Diffusion of Innovation (DOI), and Unified Theory of Acceptance and Use of Technology (UTAUT). This revision provides clarity on how these theories underpin the use of mobile health technology (see pages 3).
- **Comment 2:**- **Generalizability issue**, address limitations due to focusing on a single geographical area.
- **Response 2:-** We acknowledge the limitation of focusing on a single geographical area and have included a statement discussing the implications for generalizability in the limitations section (see page 11-12).
- **Comment 3:- Questionnaire Validation issues**, Provide more details on how the questionnaire was validated.
- **Response 3**:- We expanded on the questionnaire validation process, detailing the expert reviews conducted and adjustments made based on their feedback. This includes clarity on language and question relevance (see page 4).
- **Comment 4:- Discussion Section**: Compare findings with similar studies in other low- and middle-income countries and Discuss unexpected findings and their potential explanations.
- **Response 4**:- We have compared our findings with similar studies in low- and middle-income countries and discussed unexpected findings in more depth, including potential explanations (see pages 11-12).
- **Comment 5:- Conclusion and Recommendations**: Offer more actionable recommendations for future research and practical implementation in Ethiopia.
- **Response 5**:- Actionable recommendations for future research and practical implementation in Ethiopia have been added, focusing on improving eHealth training and infrastructure (see page 12).
- **Comment 6:- Data Availability**: Include details on data availability and any access restrictions.
- **Response 6**:- We included a detailed statement regarding data availability, specifying how interested parties can request access to the data (see page 5).
- **Comment 7:- Language and Presentation**: correct Minor issues with grammar, typographical errors, and formatting.
- **Response 7:-** We carefully reviewed the manuscript for grammatical, typographical, and formatting errors. All issues have been corrected to improve clarity and presentation.
- **Comment 8:- Ethical Considerations**: Add a statement on participants' confidentiality and data security.
- **Response 8:-** A statement on participants' confidentiality and data security has been added to the ethical considerations section (see page 5).

**Reviewer #2's Comments and Our Responses**

- **Comment 1:- Literature Review**: Needs to address gaps in the current literature, especially concerning eHealth models. Specific papers were recommended for citation.
- **Response 1**:- We have addressed gaps in the literature regarding eHealth models and included citations from the recommended papers, enhancing the relevance of our literature review (see page 3).
- **Comment 2:- Research Design** Requires clarification.
- **Response 2**:- We provided clarification regarding the research design, explaining its suitability for our study objectives (see page 4).
- **Comment 3:- Data Quality Issue**: Conduct a rigorous test of the data quality.
- **Response 3:-** We conducted additional rigorous tests of data quality, including checks for multicollinearity, residual analysis, and validation of the data entry process (see page 4).
- **Comment 4:- MLR-Based Analysis**: Reliability of the analysis needs to be addressed.
- **Response 4:-** The reliability of the analysis has been thoroughly addressed. We included details on the Variance Inflation Factor (VIF) and residual analysis to validate assumptions (see page 5).

We believe these revisions significantly enhance the clarity and rigor of our manuscript. We appreciate your guidance in this process and are hopeful that the changes meet your expectations.

Thank you for your consideration. We look forward to your feedback.

Sincerely,
Shimeles Wondimu Kebede

Lecturere at Mizan-Aman Health Science College, Department of public health

Mizan-Aman Health Science College
