# Supplementary material for: Intention to use mobile health in antenatal care service: Among primary health care unit workers, Bench-Sheko zone, southwest Ethiopia
Source: PLOS Digit Health. 2025 Apr 22;4(4):e0000587. doi: 10.1371/journal.pdig.0000587 (PMC12013934; doi:10.1371/journal.pdig.0000587)
Supplement: Attachment — Submitted filename: Response_to_Reviewers_auresp_2.docx [file pdig.0000587.s004.docx]

Shimeles Wondimu Kebede

Mizan-Aman Health Science College, Department of Public Health

Mizan-Aman City, Bench-Sheko Zone, South West Ethiopia

Shimeleswondimu@gmail.com
Date: January 26, 2025

PLOS Digital Health

Dear Editors and Reviewers,

Thank you for the opportunity to revise and resubmit our manuscript titled **"Intention to Use Mobile Health Technology in Antenatal Care Services: Among Primary Health Care Unit Workers in Bench-Sheko Zone, Southwest Ethiopia"**. We appreciate the reviewers' constructive feedback, which has significantly improved the quality of our work. Below, we provide a point-by-point response to each comment. All changes made to the manuscript are highlighted in the revised version.

**Reviewer #2's Comments and Our Responses**

1. **Abstract Overstatement**

- **Comment:** The first sentence of the abstract overstates the benefits of mobile health technology (mHealth) in maternal and child health.
- **Response:** Thank you for pointing this out. We have revised the sentence to adopt a more cautious tone, avoiding overstatements about mHealth’s impact. Please see page 2 in the revised manuscript.

2. **Cost of Mobile Technology**

- **Comment:** mHealth technologies can be expensive, posing a significant barrier in low-income settings.
- **Response:** We have expanded the discussion section to include a detailed analysis of the cost-related barriers to mHealth adoption in low-income settings. Please see page 13-14 in the revised manuscript.

3. **Use of Mobile Technology in Low-Income Settings**

- **Comment:** Discuss the limitations of implementing mHealth in settings where healthcare workers face a high patient-to-staff ratio and lack technological expertise.
- **Response:** We have added a paragraph in the discussion section addressing these limitations and emphasizing the complementary role of mHealth rather than replacing existing systems. Please see page 13-14 in the revised manuscript.

4. **Intent vs. Actual Use Graph**

- **Comment:** Include a graph comparing participants' intention to use mHealth with their actual use.
- **Response:** While the actual use of mHealth technology is a crucial metric for understanding its adoption, data on actual usage was not collected in this study. As a result, a direct comparison between participants' intention to use mHealth and their actual use could not be made. However, future studies should consider tracking actual usage through follow-up surveys or system logs to better understand this gap. We have acknowledged this limitation and recommended future studies to explore the intention-usage gap. Please see page 14 in the revised manuscript.

5. **Sample Size**

- **Comment:** Address the limitations of the sample size and data collection period.
- **Response:** We acknowledged it as a limitation and we write it in the discussion. Please see page 14 in the revised manuscript.

6. **Statistical Analysis (P-value)**

- **Comment:** Clarify the rationale for using a p-value threshold of < 0.25 for variable inclusion.
- **Response:** The manuscript now clarifies that the threshold was part of an exploratory approach for variable inclusion, with final significance set at p < 0.05. Please see page 6 in the revised manuscript.

7. **Discussion on Mobile Healthcare Limitations**

- **Comment:** Add a discussion on the limitations of mobile healthcare in low-resource settings.
- **Response:** We try to integrate a discussion of mobile healthcare limitations and current research trends and citing relevant studies to contextualize our findings. Please see page 14 in the revised manuscript.

**Reviewer #3's Comments and Our Responses**

1. **Questionnaire Development and Use**

- **Comment:** Provide more detail on how the questionnaire was developed, including the use of frameworks like TAM, DOI, and UTAUT.
- **Response:** We have expanded the methodology section to describe the development and adaptation of the questionnaire based on these frameworks. Please see page 5 in the revised manuscript.

2. **Clarity of Questionnaire Terminology**

- **Comment:** Define terms like “frequently” and clarify the response structure.
- **Response:** Key terms have been explicitly defined, and details on the response structure have been added to the methodology. Please see page 5.

3. **Background Context on Ethiopia’s Health Landscape**

- **Comment:** Provide more background on Ethiopia’s health system and telemedicine progress.
- **Response:** The introduction and discussion sections now include additional context on Ethiopia’s healthcare landscape and telemedicine policies. Please see page 3&14 in the revised manuscript.

4. **Citation Error for Saeid et al.**

- **Comment:** Correct the citation error and ensure completeness of references.
- **Response:** The reference to Saeid et al. has been corrected and updated. See the revised reference list.

5. **Figures and Tables**

- **Comment:** Evaluate the necessity of Figures 1 and 2.
- **Response:** We have removed Figures 1 and 2 to streamline the manuscript.

**Reviewer #4's Comments and Our Responses**

1. **Grammar Issues**

- **Comment:** Address grammatical errors in the results section and elsewhere.
- **Response:** The manuscript has been thoroughly proofread, and grammatical errors have been corrected.

2. **Justification for Table 2**

- **Comment:** Clarify the relevance of Table 2.
- **Response:** Table 2 has been included to provide a comprehensive overview of the behavioral intention of participants to use mHealth technology in antenatal care services. While the questions might appear similar, each one targets a specific dimension of intention, such as planning, predicting, or expressing commitment to using mHealth. This differentiation is essential for capturing subtle variations in participants' behavioral intentions. The inclusion of Table 2 allows for a nuanced understanding of these factors and supports the validity of our findings.

We hope the revised manuscript meets the reviewers’ expectations. Thank you for your time and consideration. We are happy to provide further clarifications if needed.

Sincerely,
Shimeles Wondimu Kebede

Corresponding Author
